# Development of Peptide Nucleic Acid Probes for Detection of the *HER2* Oncogene

**DOI:** 10.1371/journal.pone.0058870

**Published:** 2013-04-10

**Authors:** Belhu Metaferia, Jun S. Wei, Young K. Song, Jennifer Evangelista, Konrad Aschenbach, Peter Johansson, Xinyu Wen, Qingrong Chen, Albert Lee, Heidi Hempel, Jinesh S. Gheeya, Stephanie Getty, Romel Gomez, Javed Khan

**Affiliations:** 1 Pediatric Oncology Branch, National Cancer Institute, National Institutes of Health, Bethesda, Maryland, United States of America; 2 The Advanced Biomedical Computing Center, SAIC-Frederick, Inc., National Cancer Institute-Frederick, Frederick, Maryland, United States of America; 3 Department of Electrical and Computer Engineering, University of Maryland, College Park, Maryland, United States of America; 4 Goddard Space Flight Center, National Aeronautic and Space Administration, Greenbelt, Maryland, United States of America; Medical University Graz, Austria

## Abstract

Peptide nucleic acids (PNAs) have gained much interest as molecular recognition tools in biology, medicine and chemistry. This is due to high hybridization efficiency to complimentary oligonucleotides and stability of the duplexes with RNA or DNA. We have synthesized 15/16-mer PNA probes to detect the *HER2* mRNA. The performance of these probes to detect the *HER2* target was evaluated by fluorescence imaging and fluorescence bead assays. The PNA probes have sufficiently discriminated between the wild type *HER2* target and the mutant target with single base mismatches. Furthermore, the probes exhibited excellent linear concentration dependence between 0.4 to 400 fmol for the target gene. The results demonstrate potential application of PNAs as diagnostic probes with high specificity for quantitative measurements of amplifications or over-expressions of oncogenes.

## Introduction

There is a growing demand for rapid methods of detection for genetic aberrations that are implicated in various diseases. Such clinical tests require sensitive, reliable, cost-effective, and high throughput analytical methods. The ability to qualitatively and quantitatively detect oncogenes significantly improves accuracy of early detection, disease staging, prevention, and plan personalized therapy [1]. In this report, we developed peptide nucleic acid (PNA) probes for the detection of an oncogene known as *HER2*, which encodes a 185 kDa tyrosine kinase in the family of human epidermal growth factor receptors. It has been shown that 20–30% of breast cancer patients have amplification and over-expression of *HER2* oncogene which have been correlated with aggressive, drug resistant and poor prognosis in breast cancer [2]. These characteristics clearly establish the potential use of *HER2* as a biomarker of the disease as well as for the development of targeted and personalized treatment [3]–[5]. Currently there are two FDA approved *HER2* tests in the clinics namely; immuno-histochemistry (IHC) and fluorescence in-situ hybridization (FISH) which are mainly applied to strategize therapeutic regimen [6], [7]. However, both tests are semi-quantitative and require sophisticated laboratory techniques and instrumentation, therefore, it is desirable to develop simpler, sensitive, rapid, and scaleable genetic detection methods to check the *HER2* status of breast cancer patients [8], [9].

Efficient and robust oligonucleotide probes play a crucial role in detecting large and complex DNA/RNA molecules. The most common DNA based probes are prone to hydrolytic degradation and have lower affinity to targets under stringent hybridization conditions. Synthetic modifications of DNA molecules for example, locked nucleic acids (LNA) have produced desirable characteristics such as hydrolytic stability, higher melting temperatures and solubility and better mismatch discriminations [10]. Here we sought to assess the potential application of synthetic peptide nucleic acids (PNA) probes to detect the *HER2* gene.

**Figure 1:**
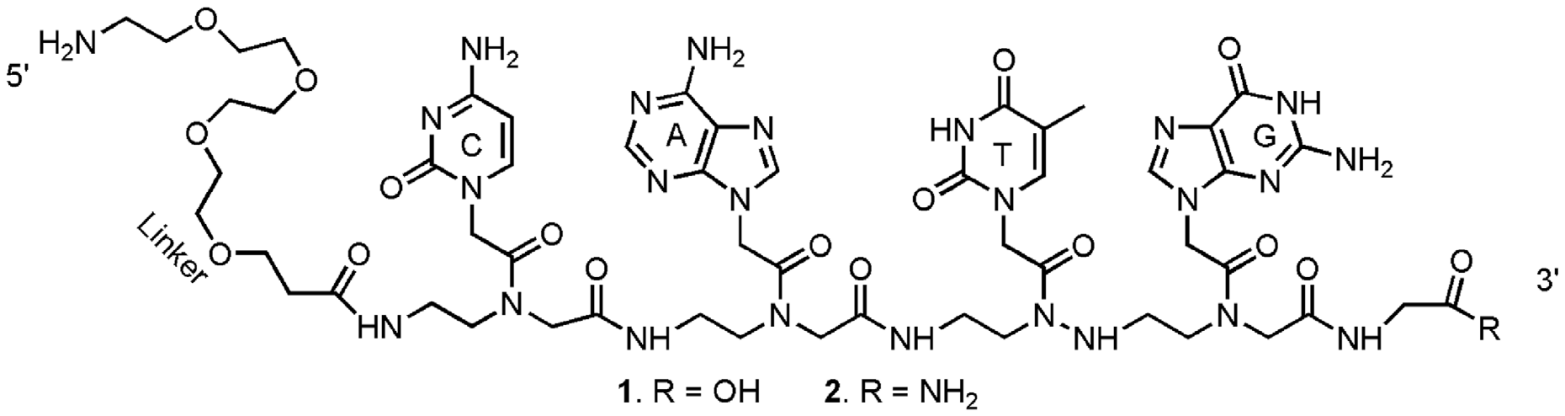
Structural representation of peptide nucleic acid (PNA) analog probes.

PNAs are synthetic DNA mimics with a repeating *N*-(2-aminoethyl)-glycine peptide neutral backbone containing purine (A,G) and pyrimidine (C,T) nucleobases [11]–[13] (Figure 1). PNA form duplexes according to the classic Watson-Crick base pairing to complimentary strands with high specificity, affinity, and greater stability compared to the corresponding DNA/DNA, RNA/DNA and RNA/RNA hybrids [14]. PNA hybridizations can be performed under low ionic strength, wide range of pH conditions [15], [16] and at relatively higher temperatures thereby providing high degree of specificity and selectivity. PNA probes are attractive due to their ability in discriminating complimentary and mismatched targets with short sequences (≤20 mer) [17]–[22] as well as their chemical stability under various conditions;. Furthermore, they are stable against hydrolytic degradation by nucleases and proteases and therefore have extended half-life in biological samples [23].

## Materials and Methods

Orthogonally *N*-protected (9-fluorenylmethoxycarbonyl (Fmoc)) and benzhydryloxycarbonyl (Bhoc) PNA monomers were purchased from Panagene (Daejeon, South Korea). MiniPEG linkers, HBTU, and Rink-Amide-MBHA resins (0.35 mmol/g) were purchased from Peptide International (Louisville, KY). Blank cartridges, reaction filters and accessories were purchased from Applied Biosystems (Foster City, CA). Color-coded carboxylated MicroPlex® assay beads were supplied by Luminex Corporation. Amine terminated glass slides (Corning® GAPS™) were obtained from Corning. All biotin labeled 25-mer DNA oligonucleotides were ordered from Integrated DNA Technologies (Coralville, IA). Single or double base mismatches (mutation) oligonucleotide were synthesized by replacing A→G, C→A, and T→G. Anhydrous solvents and reagents were purchased from Sigma-Aldrich Chemical Co. and used without purification unless otherwise mentioned. PNA synthesis was performed on Applied Biosystems 431A peptide synthesizer customized to a 5 μmol scale. Purification of all final PNA products were performed on 1200 Agilent HPLC system with heated column. MALDI spectrometry analysis was carried out on a Waters Micromass MALDI MX Spectrometer with a linear mode using 3,5-dimethoxy-4-hydroxycinnamic acid (*sinapinic acid*) solid matrix. Angiotensin II and insulin bovine with average mass of 1046.19 and 5733.58 respectively were used for mass calibration and were obtained from Sigma-Aldrich. Probe selection and melting temperatures were calculated using an in-house developed bioinformatics tool (http://pob.abcc.ncifcrf.gov/cgi-bin/PSD). PNA conjugated beads were counted using a standard light microscope and a hematocytometer. All 96 well plate fluorescence assays were performed on a Luminex*^100^* xMAP technology system [24].

### *HER2* probe design

Several 15 or 16-mer sequences from the 3′ un-translated region of the *HER2* gene were retrieved from the NCBI RefSeq database (http://www.ncbi.nlm.nih.gov/gene) that possess GC-content between 40 and 65% and melting temperatures ranging between 65 and 85°C for the DNA/PNA duplex at 1 μM concentration. The melting temperatures were calculated as described by Giesen et al., where T*_m_*_ (DNA-PNA)_  =  c_0_ + c_1_*T*_m_*_ (DNA)_ + c_2_ * # of pyrimidines + c_3_*length, where c_0_  = 20.79, c_1_  = 0.83, c_2_  = −26.13, c_3_  = 0.44 [25], [26]. The specificity of these sequences to the *HER2* transcript were checked by aligning them against the whole transcriptome and the reversed transcriptome allowing no gaps or mismatches using BLAT [27]. For sequences that have some portions of their segment hit other transcripts, the melting temperatures were calculated and compared with the melting temperature of the full 15/16-mer length. Sequences with the largest T*_m_* differences were taken as probe candidates because greater difference in melting temperature provides high hybridization affinity.

### PNA probe synthesis

**Figure 2:**
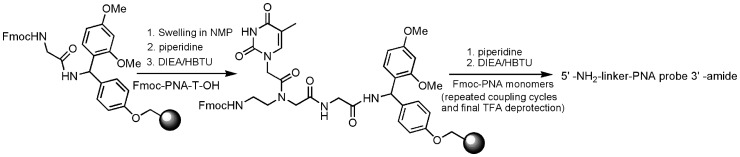
Automated synthetic scheme of PNA probes from 3′ to 5′-amino end.

PNA peptide conjugates were synthesized on a peptide synthesizer [28] customized with 125 μL delivery loop in 3 mL reaction vessel at 5 μmol scale (Figure 2). In order to prevent aggregation and maximize the synthesis of the full length PNA, the loading capacity of the resin was reduced to 0.2 mmol/g by initially reacting the resin with a glycine amino acid monomer. Monomers and linkers (Fmoc-*N*-amido-dPEG^®^3/4-acids) were weighed in to individual cartridges and pre-dissolved with 100 μL of NMP to give 0.25 M concentration and loaded onto automated synthesizer. After the synthesis (piperidine deblocking, DIEA/HBTU activation and monomer coupling cycles) is complete, the resin bound product is transferred to a polypropylene syringe tube fitted with filter and washed with NMP and treated with TFA: *m*-cresol (95∶5) for 2 to 4 h to cleave off the resin and remove all protecting groups. Most of the TFA was removed under reduced pressure by rotary evaporator and the oily residue was treated with large excess of cold diethyl ether (−20°C) and a white precipitate formed spontaneously which was separated by centrifugation and washed twice with ether. The crude product was dissolved in de-ionized water, freeze-dried and purified on HPLC with 5–40% acetonitrile/water (0.1% TFA) gradient over 60 min at 55°C of column temperature. Fractions containing the desired products mostly eluting as a single major peak (UV detection at 260 nm) were analyzed by mass spectrometry.

### Glass slide hybridization

**Figure 3:**
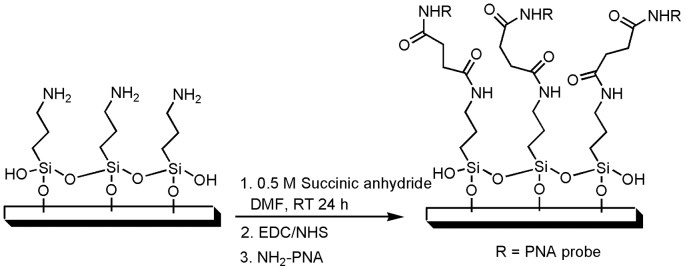
Chemistry of glass surface modification with PNA probe.

Glass slides with amine modification (Corning® GAPS™) were conjugated with PNA probe P1 following standard procedure (Figure 3). First the *ω*-amino-propylsilane is functionalized by soaking glass slides in 0.5 M solution of succinic anhydride in DMF for 24 h. After washing the slides twice with fresh DMF and twice with anhydrous dichloromethane, the slides were air dried and were immersed in a solution of DIC (0.8 M) in DMF and after 30 min solid NHS (0.8 M) was added and left over night [29], [30]. The glass slides were washed as previously described and were kept in desiccated storage box until later use. Probes were spotted on to an activated slide by applying 0.5 μL of P1 (100 μM in 1 M betaine) and left over night in a humidified chamber. The slides were then soaked in 50 mM ethanolamine for 15 min to quench all unreacted groups. After blocking, the slides were washed with de-ionized water and were ready for hybridization with the target. The 5′-biotin labeled DNA target (25 μL, 2 μM) prepared in 0.1x sodium sarcosyl (SSarc) buffer was spotted and hybridized for 2 h at 55°C. Then the slides were immediately soaked in 0.1x SSarc at 50°C and washed once more with 0.1x SSarc solution at 50°C. Streptavidin-phycoerythrin (SA-PE) in 1x TMAC was applied onto the slides and incubated for 15 min with cover-slips. The slides were then washed with 0.1x SSarc and scanned on GenePix®4000B Microarray Scanner.

### Gold surface modification

Gold surface chips (ca. 2 cm^2^) on a silica wafer were plasma cleaned and/or cleaned with piranha solution and were washed with de-ionized water and the chips were then dried under argon gas. Self assembly experiment was performed with a 5 mM 11-mercaptoundecanoic acid solution in ethanol (NanoThinks™Acid11) by immersing the cleaned chips in 4 mL of the mercapto acid for 18 h. The chips were then washed twice with ethanol and de-ionized water. The surface was activated by spotting EDC/NHS (2 μL of 40 mg/mL EDC and 2 μL of 5 mg/mL NHS) in de-ionized water and left for 10 min and treated with amine PNA probe P1 (2 μL of 50 μM) overnight. The chips were then washed with copious amount of de-ionized water and dried under argon gas. To ensure the PNA probes were attached to the surface, we used IR and X-ray photoelectron (XPS) spectroscopy to characterize the surface of gold surface chips with a Spectrum*One* FT-IR spectrometer (Perkin Elmer, Waltham, MA) and an M probe ESCA (VG Scienta, Newburyport, MA) respectively (Figure S2 and S3). Similar hybridization protocols as described for glass slides were used and fluorescence images were scanned on a flat-bed Typhoon 9410 Variable Mode Imager (GE, Piscataway, NJ).

### PNA-bead conjugation

**Figure 4:**
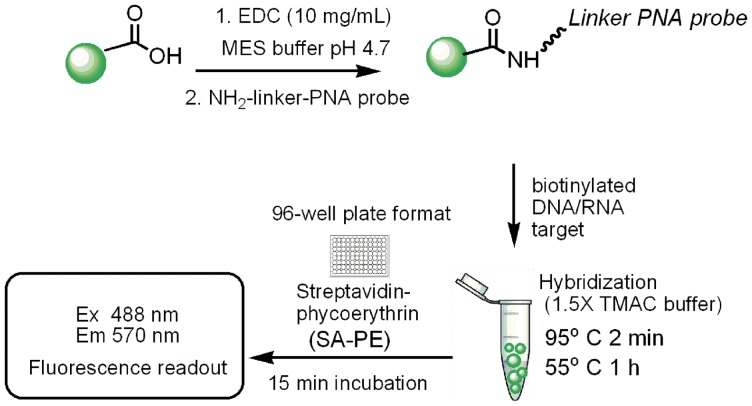
Conjugation of PNA probes and generalized scheme for the Luminex fluorescence bead assay.

Each PNA probe with 5′ free amine end was covalently coupled to uniquely color-coded carboxylated microsphere (5.6±0.1 micron, 10^8^ carboxy conjugation sites/bead) beads using a standard carbodiimide coupling chemistry (Figure 4). Briefly, one million beads were suspended with 20 μL of 2-(*N*-morpholino)-ethanesulfonic acid (MES) buffer (pH 4.7); then 10 μL of freshly dissolved 1-ethyl-3-(3-dimethylaminopropyl) carbodiimide hydrochloride (EDC) (10 mg/mL) in deionized water was added, followed by addition of 400 pmole (0.1 mM, 4 μL) of amine-terminated PNA probe. The reaction mixture was incubated in the dark for 30 minutes with occasional mixing and for an additional 30 minutes with addition of freshly prepared 10 μL of EDC. The reaction was quenched and washed with 500 μL of 0.02% Tween-20 twice and once with 0.1% SDS. The modified microspheres stock was counted and stored in 1.5x TMAC buffer at 4°C in the dark until future use.

### Fluorescence bead assays

Each color coded microsphere can be individually addressed and in principle 100 different genes can be assayed simultaneously. However, as a proof of principle we chose single color bead with PNA probe to detect the gene of interest (Figure 4). Briefly, ca. 5000 PNA conjugated beads in 33 μL 1.5x TMAC were mixed with biotinylated *HER2* DNA targets in 17 μL TE buffer with quantities ranging from 1.7 to 1700 fmol and the mixture was hybridized at 95°C (2 min) and then at 55°C (60 min) on a thermocycler. The hybridized beads were washed with 1x TMAC to remove unbound targets and then labeled with red fluorescent SA-PE (20 μg/μL in 1x TMAC) at 55°C for 15 minutes inside the heated plate holder. The average fluorescence was recorded with excitation at 488 nm and emission at 570 nm.

### Quantitative Reverse Transcription PCR (qRT-PCR)

Total RNA was reverse transcribed into cDNA using a random hexamer primer and *HER2* expression was measured by qPCR using Taqman Gene Expression Assay (Hs99999005_mH, Applied Biosystems, Foster City, CA) according to the manufacturer's protocol. Briefly, PCR reaction mixture containing Taq polymerase, specific primers and probe, and cDNA template in 1x PCR buffer was incubated at 95°C (15 sec) and 60°C (1 min) for 40 cycles in thermocycler. At the end of each cycle fluorescent signal was measured. Threshold cycle (Ct) was then determined for each samples where fluorescent signal increases exponentially. *HER2* expression is about more than 42 fold higher in SKBR3 cell lines as compared to MCF7 cell lines (with *GAPDH* normalization).

### Preparation of biotin labeled antisense *HER2* RNA target

Biotinylated antisense *HER2* RNA (aRNA) targets were prepared using GeneChip One-Cycle Target Labeling and Control Reagents (Affymetrix, Santa Clara, CA) according to the manufacturer instruction. Briefly, 5 μg of total RNA was reverse transcribed into single-stranded cDNA using a T7-oligo-(dT) primer. The single-stranded cDNA was then converted to double-stranded cDNA using cocktails of RNase H, DNA polymerase I, and DNA ligase. The double-stranded cDNA was transcribed to biotinylated aRNA using T7 RNA polymerase.

## Results and Discussion

We synthesized several PNA probes targeting the *HER2* RNA and in most cases the automated synthesis produced essentially full length PNA probes with good yields; after HPLC purification the products were characterized by mass spectrometry (Table 1). The 5′-end of the probes was modified with amine terminated mini-PEG linkers for efficient conjugation to the carboxy- modified beads, glass slides, and gold chips. Covalent attachment of PNA probes on gold chips were checked by IR spectroscopy and significant peaks were observed at 2964 (C-H stretch), 1736 (C = O stretch), 1365 (C-O and C-N bend/stretch), and 1216 cm^−1^ (C-O stretch) Surface analysis of gold chips by XPS showed significant signals for C(1 s), N(1 s), and O(1 s) further confirmed surface derivatization.

**Table 1:** *HER2* PNA probes and targets.

PNA synthetic probes	*m/z*(calcd)	*m/z*(found)	T*_m_*°C(1 μM)	Target 5′biotin-3′
P1 *NH_2_-linker*-*actggaccctagagtc*-*amide*	4527.3	4528.4	82.4	**T1 GCCCAATGAGACTCTAGGGTCCAGT**
P2 *NH_2_-linker*-*tgggaactcaagcag-Gly-amide*	4440.3	4439.6	74.7	**T2 ACCTTCCTTCCTGCTTGAGTTCCCA**
P3 *NH_2_-linker*-*caaaggcaaaaacgt-Gly-amide*	4418.3	4417.1	70.5	**T3 GTCGTCAAAGACGTTTTTGCCTTTG**
P4 *NH_2_-linker*-*ccagtaatagaggttg-Gly-amide*	4721.5	4721.4	70.9	**T4 AGCCTTCGACAACCTCTATTACTGG**
P5 *NH_2_-linker*-*gtgtcaagtactcggg-Gly-amide*	4713.5	4715.1	80.7	**T5 GTGGAGAACCCCGAGTACTTGACAC**
P6 *NH_2_*-*linker-gactctaggtccagt-amide*	4558.4	4559.7	76.0	**T6 GTGGCATCCACTGGACCCTAGAGTC**
P6 *NH_2_*-*linker-gactctaggtccagt-amide*	4558.4	4559.7	76.0	**cRNA**

**Figure 5:**
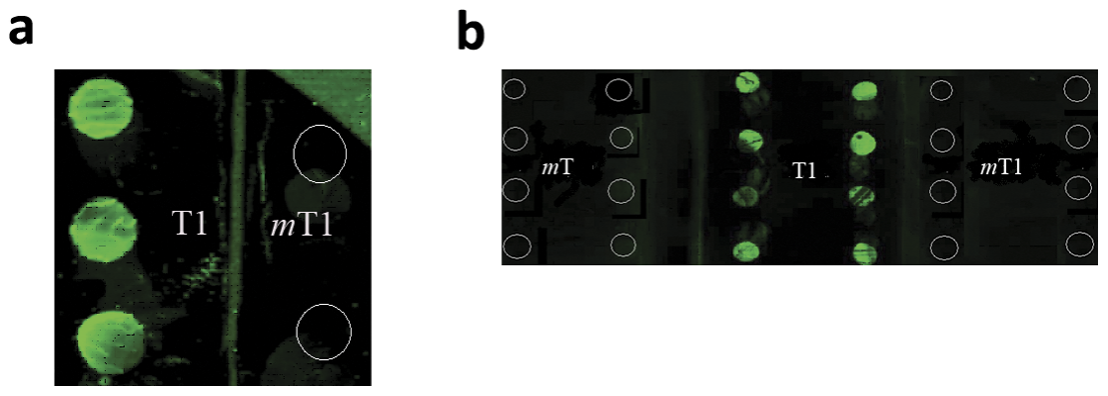
Hybridization on gold surface (a) or glass surface (b). PNA probe P1 was conjugated to the gold or glass surface at circled area. Hybridization was performed with either 50 pmol T1 (perfect match wild type target, green circles), mT (single mismatch on only glass surface, black circles,or *m*T1 (two bases mismatch, black circles) DNA targets tagged with FITC. Image was acquired after washing the targets off the surface.

Initial qualitative experiments were carried out on glass slides and gold chips modified with the P1 PNA probe. Hybridizations with a 25-mer biotin labeled synthetic DNA target T1 (50 pmol, 5′-biotin-GCCCAATGAGACTCTAGGGTCCAGT) at room temperature and 35°C produced high background fluorescence. However examination of higher temperature up to 60°C gave very good discrimination between wild type targets and mismatched targets. Furthermore, a quantitative fluorescence bead assay at 55°C gave better discrimination as compared to hybridizations at 45°C (Figures S5, S6). As a result, 55°C was chosen as an optimum hybridization temperature for all subsequent hybridizations. The results from the hybridization of P1 with T1 DNA target showed strong fluorescence signals for both the glass and gold chips (Figure 5). In contrast, hybridization with two-base mismatched target *m*T1 (5′-biotin-GCCCAATGAGACTCTA*tt*GTCCAGT) produced no detectable fluorescence in either gold or glass slides. Furthermore, no fluorescence was observed with a single base mismatched target *m*T (5′-biotin-GCCCAATGAGACTCTAG*t*GTCCAGT) on glass slide hybridization (Figure 5b). These results clearly demonstrate that PNA probes sufficiently discriminate between wild type and single base mutant targets under stringent hybridization conditions. With this background, we continued to quantitatively assess the efficiency of the PNA probes with fluorescence bead assays.

For quantitative measurements we next used the Luminex bead system on which the probes were covalently attached (see Figure 4 and methods). First, the density of probe coverage was optimized by conjugating five different amounts (0.2, 0.4, 0.6, 0.8, 1.0 nmol per 106 beads) of PNA probe P1. Each bead set with P1 probe was analyzed by hybridizing with a fixed amount of (17 fmol) *HER2* DNA target T1 in TE buffer. The fluorescence intensity increased linearly with increasing probe quantity, however the intensity decreased with increasing the amount of the probe higher than 0.6 nmol. The decrease in fluorescence is likely due to aggregation and inefficient hybridization to the target DNA (Figure S4). Even though the highest fluorescence intensity was recorded at about 0.6 nmol probe, probe coverage with about 0.4 nmol produced low background signal and a wide analytical linear range. We therefore decided to use 0.4 nmol/10^6^ of PNA probe amount for all subsequent bead conjugations.

Once the conditions for probe coverage were optimized, each probe was tested against its wild type DNA target and mutant type target with eight serial concentrations ranging from 1.7 fmol to 1.02 pmol. Interestingly PNA probes P1, P3, P4 and P5 gave excellent linear concentration dependence for the concentration range investigated (see supporting information, Figures S7, S9, S10, and S11 respectively). However PNA probe P2 exhibited high background fluorescence and poor linear dependence on concentration probably due to sticky sequences both in the PNA and the DNA target (Figure S8). At higher concentration (above 170 fmol) P3 and P4 showed saturation of fluorescence signal (Figure S9 and S10 respectively).

**Figure 6:**
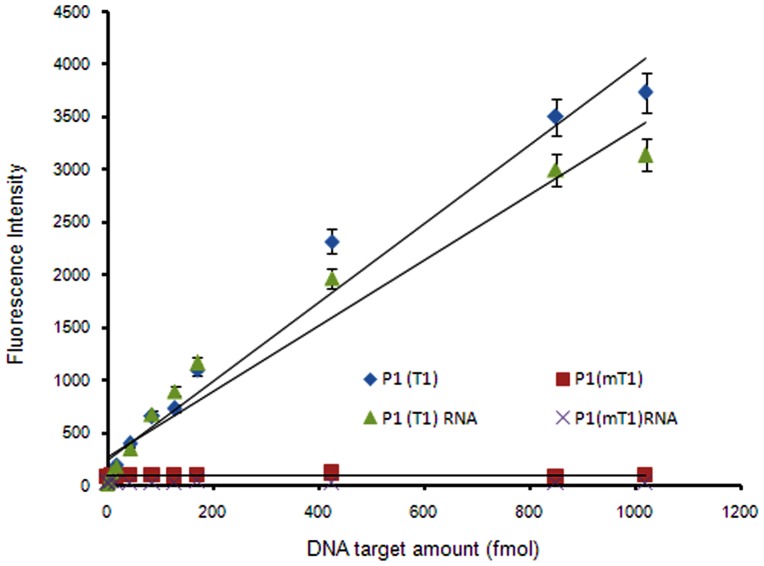
Fluorescence bead assay of T1 and *m*T1 *HER2* targets with extraneous RNA background using P1 probe.

We next examined the selectivity of these probes in complex biological matrix that could mimic clinical samples. To achieve such complexity, each DNA target sample was mixed with 1.50 μg of total RNA extracted from an Rh30 cell line (a rhabdomyosarcoma cell line purchased from ATCC) which does not express *HER2*. Measurements of target DNA in total RNA background with P1 probe gave high selectivity and specificity similar to that is obtained from measurements in buffer solutions for both the wild and mutant type targets (Figure 6). The limit of detection (LOD) was determined by measuring ten identical samples with 0.85 fmol of the target. Based on the standard deviations of these measurements, the limit of detection for P1 probe is determined to be 0.39 fmol. These results suggest the potential applications of PNA probes for analytical determination of RNA targets in complex biological samples.

**Figure 7:**
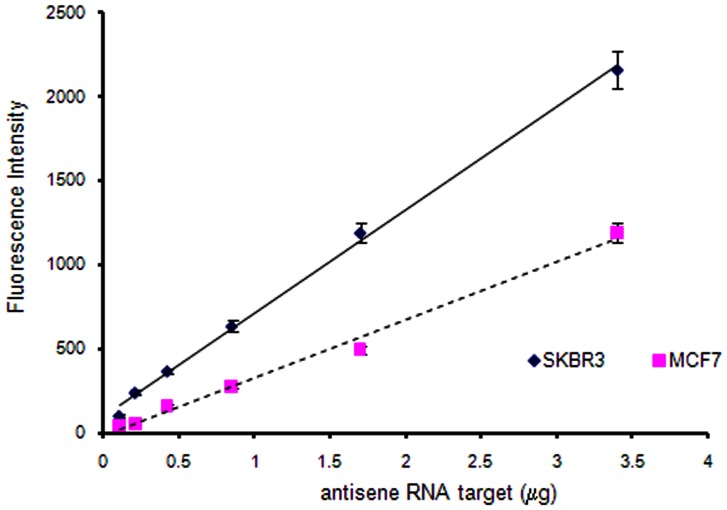
Fluorescence bead assay of *HER2* cRNA from SKBR3 and MCF7 cell lines with PNA probe P6.

Furthermore, the ability of these probes to detect and measure long chain RNAs was investigated. Biotin labeled antisense *HER2* RNA (cRNA) samples were synthesized via cDNA from total RNA extracted from MCF7 and SKBR3 (ATCC) breast cancer cell lines. These cell lines were chosen due to their differential expression or amplification of *HER2* with significantly higher expression for SKBR3 cell lines as confirmed by RT-qPCR experiment ( Figure S1). A sense PNA probe P6 (NH_2_-linker-GACTCTAGGGTCCAGT-3′) was used to detect the cRNA. Interestingly this PNA probe detected the difference in levels of *HER2* expression between SKBR3 and MCF7 cell lines (Figure 7). The analytical linear concentration ranges between 0.1 to 3.5 μg of total cRNA; this is a promising result because the cRNA sample is a very long chain compared to the 16-mer PNA probe and closely represents the complexity of real RNA sample.

## Conclusions

PNAs are versatile synthetic genetic probes due to their high affinity hybridization to complimentary sequences, chemical stability and accessibility through standard chemical synthesis. Our results demonstrate that the designed PNA probes are efficient in discriminating single and double base mismatches under stringent hybridization conditions. The probes have shown very good sensitivity and specificity in complex RNA matrices which are important parameters for direct detection and analysis of mRNA in biological fluids. The observed analytical linear range (0.4–400 fmol) is sufficiently large and show great potential for both qualitative and quantitative analysis of genetic mutations and expression levels. Direct and label-free detection methods could be developed by integrating these PNA probes with field effect transistors [21], [31]. Such detection methods would result in a highly sensitive, reliable and compact instrumentation that is widely available in routine testing and monitoring.

## Supporting Information

Figure S1 **qRT-PCR measurement of** ***HER2*** **levels in MCF7 and SKBR3 cancer cell line.**
(PDF)

Figure S2 **IR spectrum of PNA modified gold surface.**
(PDF)

Figure S3 **XPS spectra of PNA modified gold surface.**
(PDF)

Figure S4 **Measurements of** ***HER2*** **DNA targets with PNA probes.**
(PDF)

Figure S5 **Bead based hybridization assay of T6 (antisense) target with PNA probe P6 at 45°C.**
(PDF)

Figure S6 **Bead based hybridization assay of T6 (antisense) target with PNA probe P6 at 55°C.**
(PDF)

Figure S7 **Fluorescence bead assay of DNA target T1 with** ***HER2*** **PNA probe P1.**
(PDF)

Figure S8 **Fluorescence bead assay of DNA target T2 with** ***HER2*** **PNA probe P2.**
(PDF)

Figure S9 **Fluorescence bead assay of DNA target T3 with** ***HER2*** **PNA probe P3.**
(PDF)

Figure S10 **Fluorescence bead assay of DNA target T4 with** ***HER2*** **PNA probe P4.**
(PDF)

Figure S11 **Fluorescence bead assay of DNA target T5 with** ***HER2*** **PNA probe P5.**
(PDF)

## References

[1] Rasooly A, Jacobson J (2006) Development of biosensors for cancer clinical testing. Biosensors and Bioelectronics 21: 1851–1858. 16458498 10.1016/j.bios.2006.01.003

[2] Salmon DJ, Clark GM, Wong SG, Levin WJ, Ullrich A, et al (1987) Human breast cancer: Correlation of relapse and survival with amplification of the HER2/neu oncogene. Science 235: 177–182. 3798106 10.1126/science.3798106

[3] Yu D, Liu B, Jing T, Sun D, Price JE, et al (1998) Overexpression of both p185c-erbB2 and p170mdr-1 renders breast cancer cells highly resistant to taxol. Oncogene 16: 2087–2094. 9572489 10.1038/sj.onc.1201729

[4] Knuefermann C, Lu Y, Liu B, Jin W, Liang K, et al (2003) HER2/PI-3K/Akt activation leads to a multidrug resistance in human breast adenocarcinoma cells. Oncogene 22: 3205–3212. 12761490 10.1038/sj.onc.1206394

[5] Stark A, Hulka BS, Joens S, Novotny D, Thor AD, et al (2000) HER2/neu amplification in benign breast disease and the risk of subsequent breast cancer. J Clin Oncol 18: 267–274. 10637239 10.1200/JCO.2000.18.2.267

[6] Owens MA, Horten BC, Da Silva MM (2004) HER2 amplification ratios by fluorescence in situ hybridization and correlation with immune-histochemistry in a cohort of 6556 breast cancer tissues. Clin Breast Cancer 5: 63–69. 15140287 10.3816/cbc.2004.n.011

[7] Yaziji H, Goldstein LC, Barry TS, Werling R, Hwang H, et al (2004) HER2 testing in breast cancer using parallel tissue-based methods. J Am Med Assoc 291: 1972–1977. 10.1001/jama.291.16.1972 15113815

[8] Noske A, Loibi S, Darb-Esfhani S, Roller M, Kronenwett R, et al (2011) Comparison of different approaches for assessment of HER2 expression on protein and mRNA level. Breast Cancer Res Treat 126: 109–117. 21190079 10.1007/s10549-010-1316-y

[9] Winston JS, Ramanaryanan J, Levine E (2004) HER2/neu evaluation in breast cancer. Am J Clin Pathol 121 (Suppl 1)S33–S49. 15298149 10.1309/9UNL7UXPYO6CPWBQ

[10] You Y, Moreira BG, Behlke MA, Owczarzy R (2006) Design of LNA probes that improves mismatch discrimination. Nucleic Acid Res 34: e60. 16670427 10.1093/nar/gkl175 PMC1456327

[11] Nielsen PE, Egholm M, Berg RH, Buchardt O (1991) Sequence-selective recognition of DNA by strand displacement with a thymine-substituted polyamide. Science 254: 1497–1500. 1962210 10.1126/science.1962210

[12] Egholm M, Buchardt O, Nielsen PE, Berg RH (1992) Peptide nucleic acids (PNA). Oligonucleotide analogs with an achiral peptide backbone. J Am Chem Soc 114: 1895–1897.

[13] Buchardt O, Christensen L, Behrens C, Freier SM, Driver DA, et al (1993) PNA hybridizes to complimentary oligonucleotides obeying the Watson-Crick hydrogen-bonding rules. Nature 365: 566–568. 7692304 10.1038/365566a0

[14] Kaihatsu K, Braasch DA, Cansizoglu A, Corey DR (2002) Enhanced strand invasion by peptide nucleic acid-peptide conjugates. Biochemistry 41: 11118–11125. 12220176 10.1021/bi0263659

[15] Jensen K, Orum H, Nielsen PE, Norden B (1997) Kinetics for hybridization of peptide nucleic acids (PNA) with DNA and RNA studied with the BIAcore technique. Biochemistry 36: 5072–5077. 9125529 10.1021/bi9627525

[16] Schwarz FP, Robinson S, Butler JM (1999) Thermodynamic comparison of PNA/DNA and DNA/DNA hybridization reactions at ambient temperature. Nucleic Acid Res 27: 4792–4800. 10572180 10.1093/nar/27.24.4792 PMC148780

[17] Pellestor F, Paulasova P (2004) The peptide nucleic acids (PNAs), powerful tools for molecular genetics and cytogenetics. Eur J Human Gen 12: 694–700. 10.1038/sj.ejhg.5201226 15213706

[18] Yoshiaki N, Huqun HM, Tanaka T, Udagawa K, Kato M, et al (2005) Genetic heterogeneity of the epidermal growth factor receptor in non–small cell lung cancer cell lines revealed by a rapid and sensitive detection system, the peptide nucleic acid-locked nucleic acid PCR clamp. Cancer Res 65: 7276–7282. 16105816 10.1158/0008-5472.CAN-05-0331

[19] Robertson KL, Yu L, Armitage BA, Lopez J, Peteanu LA (2006) Fluorescent PNA probes as hybridization labels for biological RNA. Biochemistry 45: 6066–6074. 16681379 10.1021/bi052050s

[20] Zhang N, Appella DH (2007) Colorimetric detection of anthrax DNA with a peptide nucleic acid sandwich-hybridization assay. J Am Chem Soc 129: 8424–8425. 17569540 10.1021/ja072744j

[21] Uno T, Tabata H, Kawai T (2007) Peptide-nucleic acid-modified ion-sensitive field-effect transistor-based biosensor for direct detection of DNA hybridization. Anal Chem 79: 52–59. 17194121 10.1021/ac060273y

[22] Jang H, Kim J, Choi JJ, Son Y, Park H (2010) Peptide nucleic acid array for detection of point mutations in hepatitis B virus associated with antiviral resistance. J Clin Microbiology 48: 3127–3131. 10.1128/JCM.02058-09 PMC2937715 20573874

[23] Demidov VV, Potaman VN, Frank-Kamenetskii MD, Egholm M, Buchard O, et al (1994) Stability of peptide nucleic acids in human serum and cellular extracts, Biochem Pharmacol. 48: 1310–1313. 10.1016/0006-2952(94)90171-6 7945427

[24] Dunbar SA (2006) Applications of Luminex® xMAP™ technology for rapid, high-throughput multiplexed nucleic acid detection. Anal Chimica Acta 363: 71–82. 10.1016/j.cccn.2005.06.023 PMC7124242 16102740

[25] Giesen U, Kleider W, Berding C, Geiger A, Orum H, et al (1998) A formula for thermal stability (T_m_) prediction of PNA/DNA duplexes. Nucleic Acid Res 26: 5004–5006. 9776766 10.1093/nar/26.21.5004 PMC147916

[26] Takiya T, Seto Y, Yasuda H, Suzuki T, Kawai K (2004) An empirical approach for thermal stability (T_m_) prediction of PNA/DNA duplexes. Nucleic Acid Symposium Ser 48: 131–132. 10.1093/nass/48.1.131 17150513

[27] Kent WJ (2002) BLAT – the BLAST-like alignment tool. Genome Res 12: 656–64. 11932250 10.1101/gr.229202 PMC187518

[28] Christensen L, Fitzpatrick R, Gildea B, Petersen KH, Hansen HF, et al (1995) Solid phase peptide nucleic acid synthesis. J Peptide Sci 3: 175–183. 10.1002/psc.310010304 9222994

[29] Lesaicherre ML, Uttamchandani M, Chen GYJ, Yao SQ (2002) Developing site-specific immobilization strategies of peptide microarray. Bioorg Med Chem Lett 12: 2079–2083. 12127508 10.1016/s0960-894x(02)00379-7

[30] Lee MR, Shin I (2005) Facile preparation of carbohydrate microarrays by site-specific covalent immobilization of unmodified carbohydrates on hydrazide-coated glass slides. Org Lett 7: 4269–4272. 16146404 10.1021/ol051753z

[31] Pandana H, Aschenbach KH, Lenski DR, Fuhrer MS, Khan J, et al (2008) A versatile biomolecular charge-based sensor using oxide-gated carbon nanotube transistor arrays. IEEE Sensors J 8: 655–660.

