# Supplementary figures and images for: Development of Peptide Nucleic Acid Probes for Detection of the HER2 Oncogene
Source: PLoS One. 2013 Apr 10;8(4):e58870. doi: 10.1371/journal.pone.0058870 (PMC3622650; doi:10.1371/journal.pone.0058870)

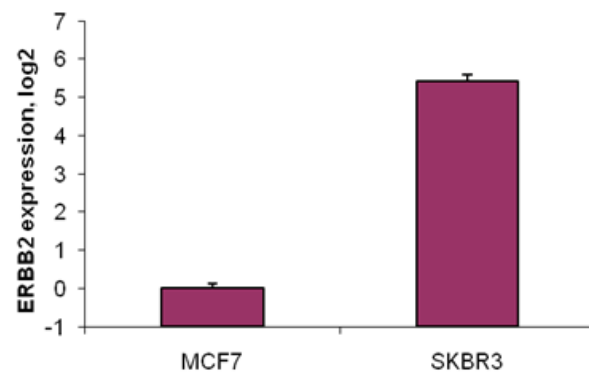

Figure S1. qRT-PCR measurement of *HER2* levels in MCF7 and SKBR3 cancer cell line

Supplement: Figure S1 — qRT-PCR measurement of HER2 levels in MCF7 and SKBR3 cancer cell line. (PDF) [file pone.0058870.s001.pdf]

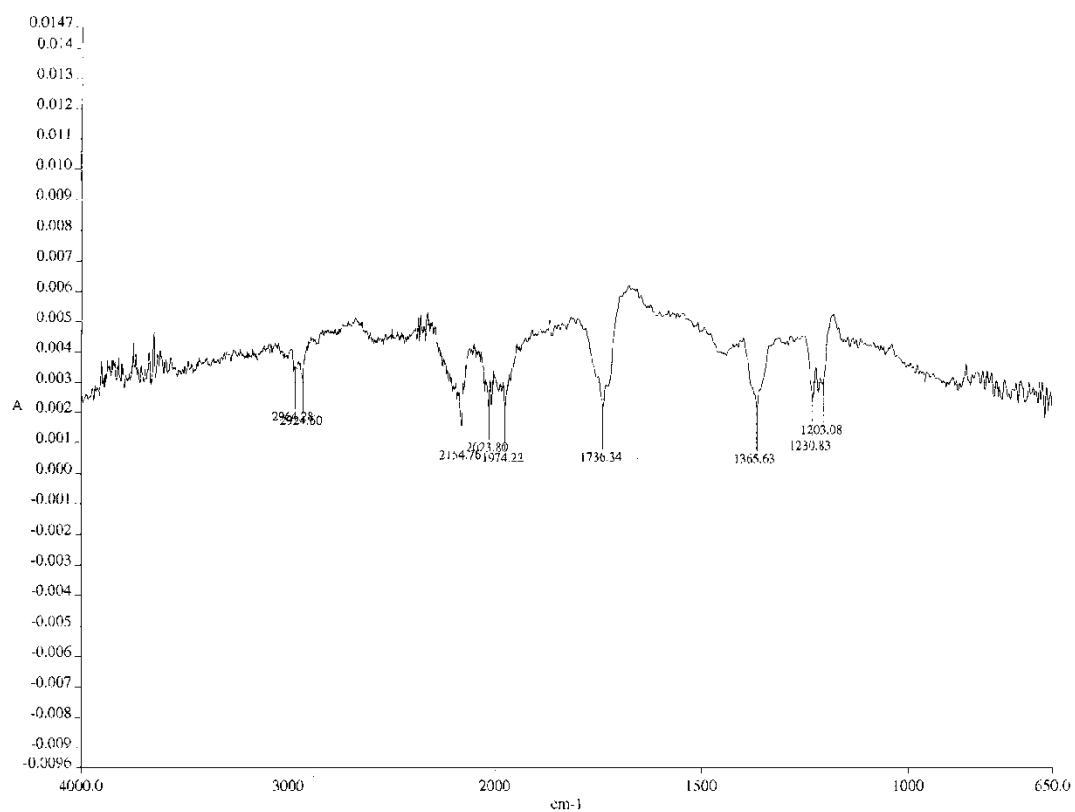

Figure S2. IR spectrum of PNA modified gold surface

Supplement: Figure S2 — IR spectrum of PNA modified gold surface. (PDF) [file pone.0058870.s002.pdf]

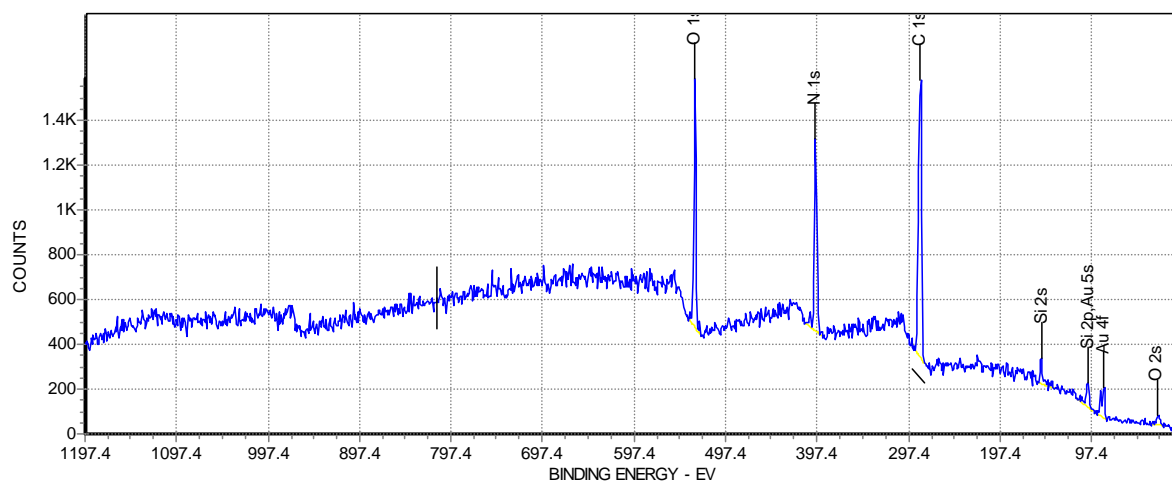

Figure S3. XPS spectra of PNA modified gold surface

Supplement: Figure S3 — XPS spectra of PNA modified gold surface. (PDF) [file pone.0058870.s003.pdf]

PNA probe P1 loading on beads (ca.  $10^6$  beads)

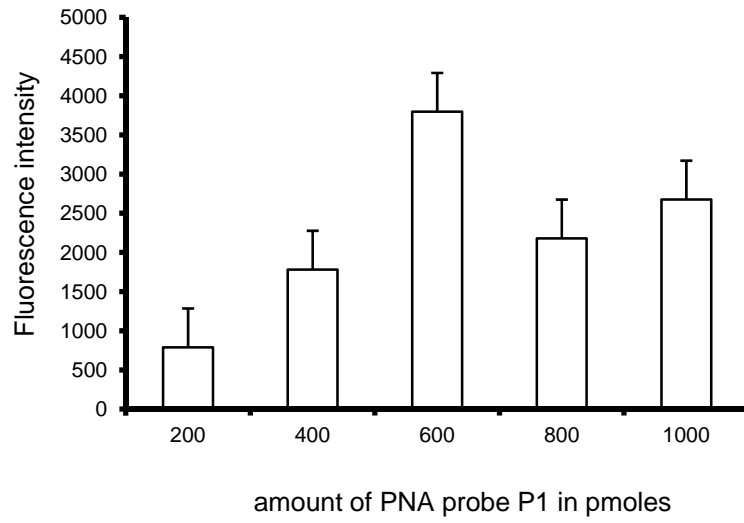

Figure S4. Measurements of *HER2* DNA targets with PNA probes

Supplement: Figure S4 — Measurements of HER2 DNA targets with PNA probes. (PDF) [file pone.0058870.s004.pdf]

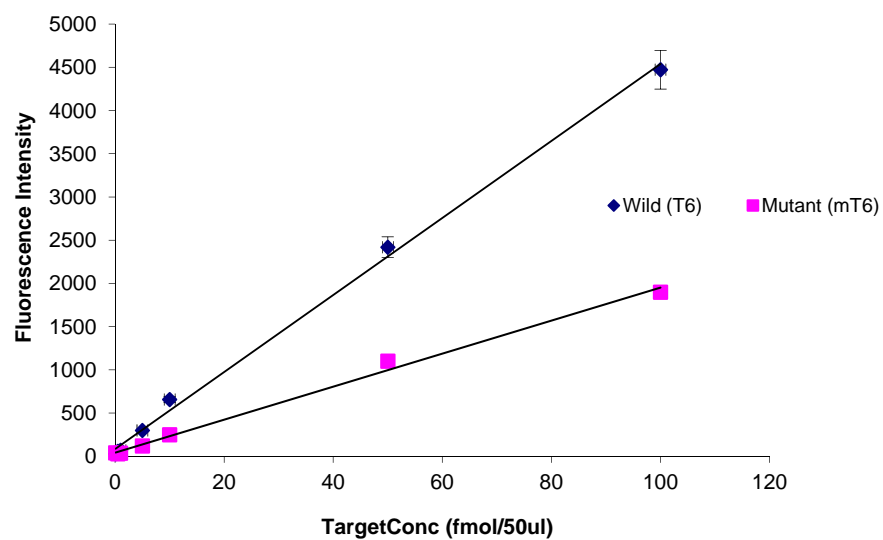

Figure S5 Bead based hybridization assay of T6 (antisense) target with PNA probe P6 at 45 °C

Supplement: Figure S5 — Bead based hybridization assay of T6 (antisense) target with PNA probe P6 at 45°C. (PDF) [file pone.0058870.s005.pdf]

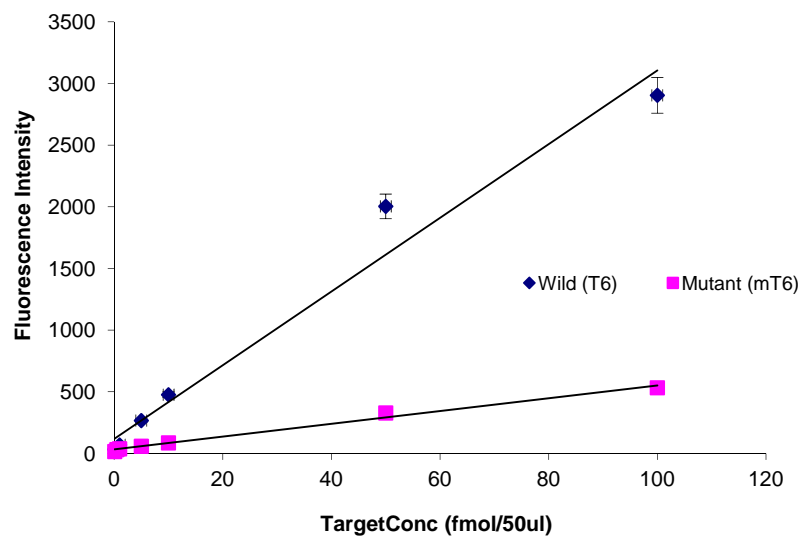

Figure S6. Bead based hybridization assay of T6 (antisense) target with PNA probe P6 at 55 °C

Supplement: Figure S6 — Bead based hybridization assay of T6 (antisense) target with PNA probe P6 at 55°C. (PDF) [file pone.0058870.s006.pdf]

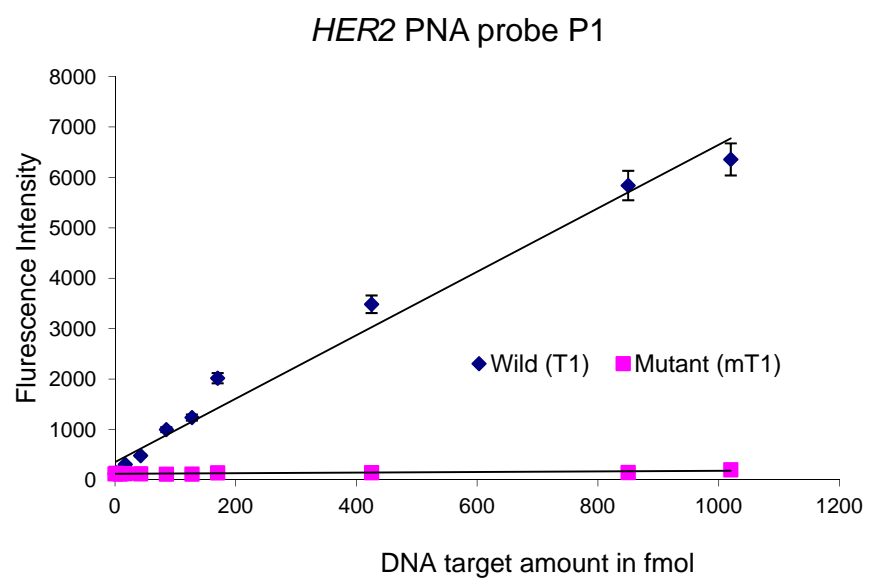

Figure S7. Fluorescence bead assay of DNA target T1 with *HER2* PNA probe P1

Supplement: Figure S7 — Fluorescence bead assay of DNA target T1 with HER2 PNA probe P1. (PDF) [file pone.0058870.s007.pdf]

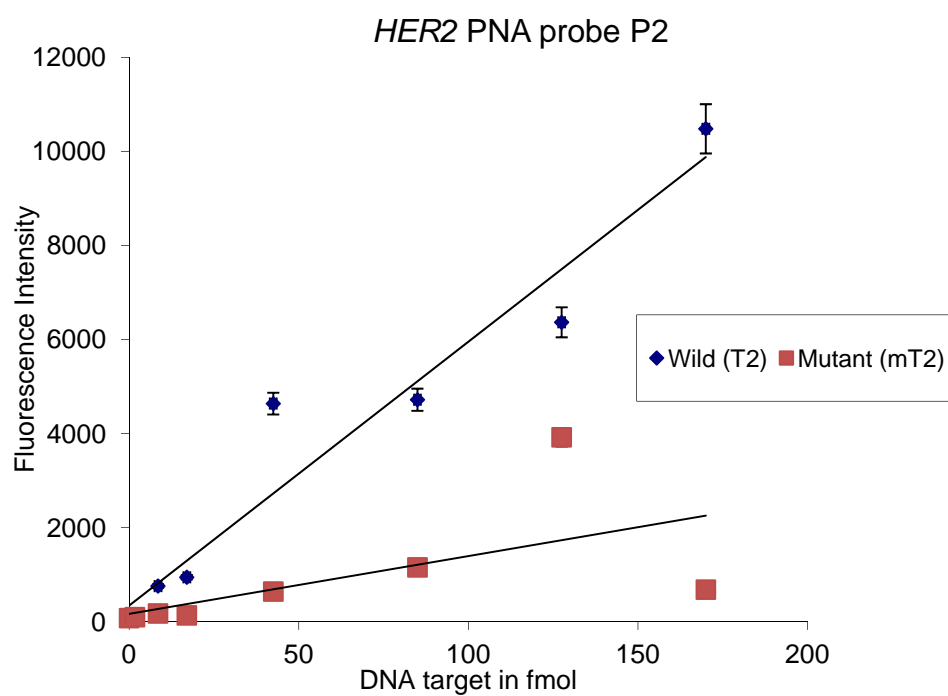

Figure S8. Fluorescence bead assay of DNA target T2 with *HER2* PNA probe P2

Supplement: Figure S8 — Fluorescence bead assay of DNA target T2 with HER2 PNA probe P2. (PDF) [file pone.0058870.s008.pdf]

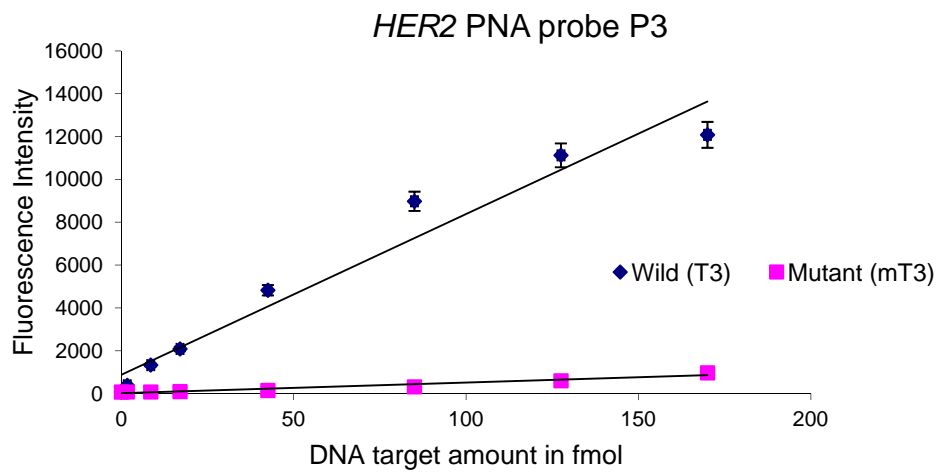

Figure S9. Fluorescence bead assay of DNA target T3 with *HER2* PNA probe P3

Supplement: Figure S9 — Fluorescence bead assay of DNA target T3 with HER2 PNA probe P3. (PDF) [file pone.0058870.s009.pdf]

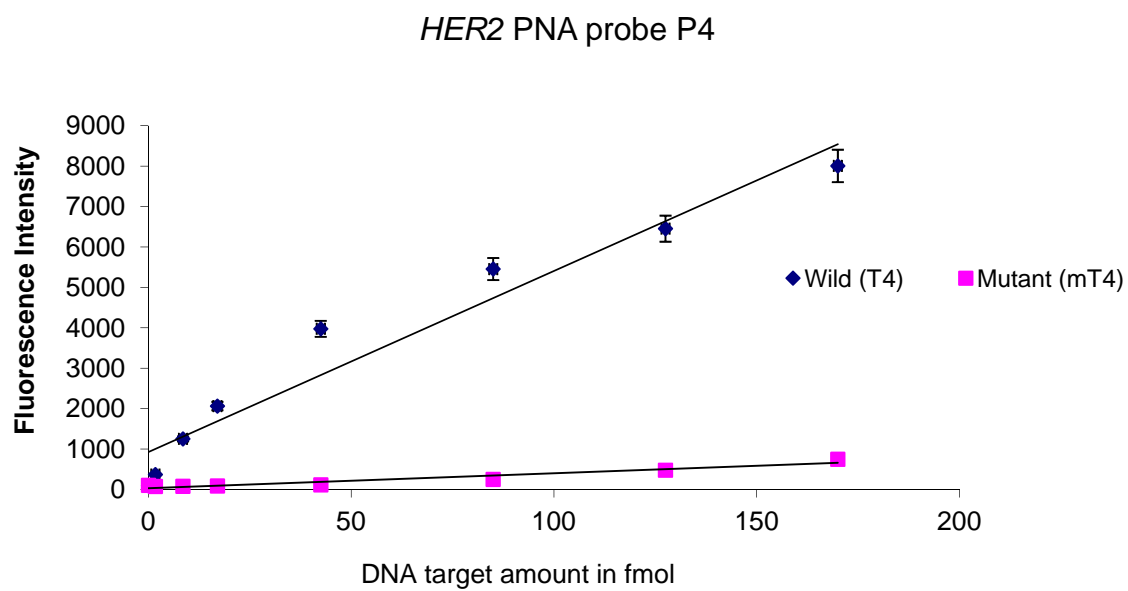

Figure S10. Fluorescence bead assay of DNA target T4 with *HER2* PNA probe P4

Supplement: Figure S10 — Fluorescence bead assay of DNA target T4 with HER2 PNA probe P4. (PDF) [file pone.0058870.s010.pdf]

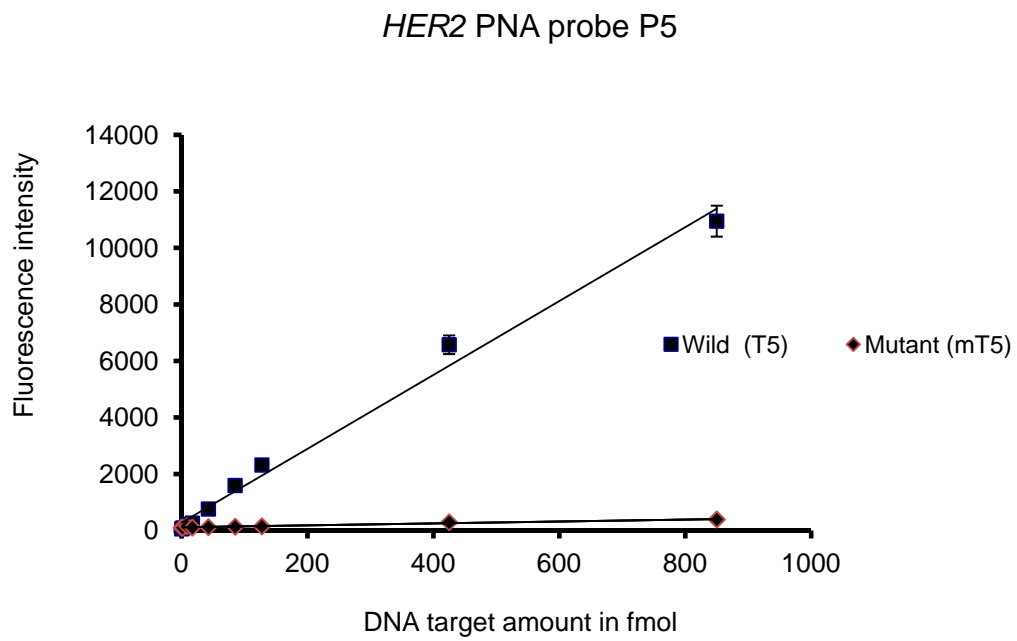

Figure S11. Fluorescence bead assay of DNA target T5 with *HER2* PNA probe P5

Supplement: Figure S11 — Fluorescence bead assay of DNA target T5 with HER2 PNA probe P5. (PDF) [file pone.0058870.s011.pdf]
